# Influence of Polypyrrole on Phosphorus- and TiO_2_-Based Anode Nanomaterials for Li-Ion Batteries

**DOI:** 10.3390/nano14131138

**Published:** 2024-07-02

**Authors:** Chiwon Kang, Kibum Song, Seungho Ha, Yujin Sung, Yejin Kim, Keun-Young Shin, Byung Hyo Kim

**Affiliations:** 1 Department of Materials Science and Engineering, Soongsil University, Seoul 06978, Republic of Korea; cwkang@ssu.ac.kr (C.K.);; 2 Department of Green Chemistry and Materials Engineering, Soongsil University, Seoul 06978, Republic of Korea

**Keywords:** phosphorus, polypyrrole, titanium dioxide, Li-ion battery, anode, nanocomposite

## Abstract

Phosphorus (P) and TiO_2_ have been extensively studied as anode materials for lithium-ion batteries (LIBs) due to their high specific capacities. However, P is limited by low electrical conductivity and significant volume changes during charge and discharge cycles, while TiO_2_ is hindered by low electrical conductivity and slow Li-ion diffusion. To address these issues, we synthesized organic–inorganic hybrid anode materials of P–polypyrrole (PPy) and TiO_2_–PPy, through in situ polymerization of pyrrole monomer in the presence of the nanoscale inorganic materials. These hybrid anode materials showed higher cycling stability and capacity compared to pure P and TiO_2_. The enhancements are attributed to the electrical conductivity and flexibility of PPy polymers, which improve the conductivity of the anode materials and effectively buffer volume changes to sustain structural integrity during the charge and discharge processes. Additionally, PPy can undergo polymerization to form multi-component composites for anode materials. In this study, we successfully synthesized a ternary composite anode material, P–TiO_2_–PPy, achieving a capacity of up to 1763 mAh/g over 1000 cycles.

## 1. Introduction

The high performance of Li-ion batteries (LIBs) strongly depends on high-quality anode materials that possess high specific capacity and long cycling stability. Graphite anode materials have been currently used for commercially available LIBs, but they have a low specific theoretical capacity. Post-graphite anodes materials for LIBs have been developed to overcome this limitation. These anode materials are categorized to intercalation-type and conversion-type anodes based on their charging mechanisms. Phosphorus (P)-based nanoscale materials, such as red phosphorus or black phosphorus, have been applied as representative intercalation-type anode materials. Nanoscale metal oxides are widely utilized as conversion-type anode materials, whose mechanism involves converting from MO_x_ to LiO_y_ and pure metal. Among the metal oxide materials, TiO_2_ has multiple merits, including long-term cycling stability, high safety, low cost, and minimal environmental impact [1]. Despite these advantages, both representative anode materials of each type, P and TiO_2_, have inherent limitations in conductivity and long-term stability. P exhibits lower electrical conductivity (~10^−10^ S/cm), higher volumetric variation (~490%) during cycling, and intermediate-species-induced shuttling effects of Li_x_P, leading to the sluggish lithiation reaction kinetics and pulverization problem [2,3]. TiO_2_ also has some drawbacks, such as low electrical conductivity (10^−12^ S/cm) and slow Li^+^ ion diffusion [4]. 

Recently, the conductivities of representative post-graphite anode materials have been improved by incorporating conducting polymers into P and TiO_2_. Conducting polymers possess unique properties, including excellent electronic conductivity [5]. The polymer chains can form a matrix that provides conductive backbones for the electrode materials of LIBs. Additionally, the structural flexibility of polymer chains allow them to serve as a host matrix, preventing large volumetric changes during charge/discharge cycles [6]. Therefore, the incorporation of a thin and robust conducting polymer coating is a simple and feasible way to enhance the electrochemical performance of P and TiO_2_ anodes. Surface coating of anodes using conducting polymer materials, such as polyaniline (PANI) [7], poly (3,4-ethylenedioxythiophene) (PEDOT) [2] and polypyrrole (PPy) [8], has been considered as an effective strategy to enhance the cycling stability of electrode materials for energy storage devices. Among these, PPy shows excellent electrical conductivity because the extra electrons forming inner double bonds can move easily through the polymer chain (10–100 S/cm), along with having outstanding chemical stability [9,10]. During the synthesis of the nanocomposite, PPy self-assembles with nanostructured P and TiO_2_, resulting in the formation of well-defined nanostructures [11,12]. PPy also can be easily polymerized from the pyrrole monomer through chemical polymerization. Hence, PPy coating has been regarded as an effective way to enhance the electrochemical properties of electrode materials, such as MnO_2_ [13], Si [6], α-Fe_2_O_3_ [14], Ti_3_C_2_T*_x_* MXene [15], FeS_2_ [16], and LiNi_0.5_Co_0.2_Mn_0.3_O_2_ (NCM) [17]. The advantages of PPy chains allow them to serve as a conducting layer on the surface of electrode particles, improving the electrical conductivity and cycling stability of the electrodes during cycling [18]. Additionally, PPy can inhibit the dissolution of phosphorus intermediates (Li_x_Ps) and their shuttle effect due to its strong chemical adsorption, thus avoiding the loss of active material P and consequently enhancing cycling stability [3]. Nevertheless, there have been few reports explaining the rationale behind the increase in specific capacity during the long-term cycling of PPy-integrated P and TiO_2_ nanocomposite anode materials. 

In this study, we prepared P–PPy and TiO_2_–PPy through two-step methods: high-energy ball milling followed by PPy polymerization, and sol–gel followed by PPy polymerization. The PPy-based composite anodes exhibited drastically enhanced LIB performance. Notably, the capacity of P–PPy and TiO_2_–PPy increased with cycles, primarily attributed to the high conductivity and structural buffering effect of PPy. We also successfully synthesized three-component hybrid anode materials, P–TiO_2_–PPy, which show excellent cycling stability with high specific capacity. Importantly, the conductive and flexible PPy coating layer not only improves electrical conductivity for the dual active anode materials, P and TiO_2_, but also prevents structural pulverization and direct contact between the active materials and the electrolyte, consequently mitigating side reactions at the solid electrolyte interface (SEI). 

## 2. Materials and Methods

### 2.1. Materials

Iron (III) chloride hexahydrate (FeCl_3_·6H_2_O), nitric acid (70%), pyrrole (98%), red phosphorus (RP, 97%), titanium (IV) isopropoxide (TTIP, 97%), and urea (98%) were purchased from Sigma-Aldrich (St. Louis, MO, USA). Absolute ethanol (99.9%) was purchased from Samchun Chemicals (Seoul, Republic of Korea).

### 2.2. Synthesis of Urea-Functionalized Phosphorus (P)

A planetary ball mill was used to facilitate the formation of P-based nanoscale materials through the functionalization of urea. Initially, red phosphorus (RP, 0.5 g) and urea (1 g) were placed in a ball milling jar with 1 mm diameter zirconia balls at a ratio of 1:500. Ball milling was carried out for 24 h at 550 rpm, with the process paused for 5 min every 24 min to prevent overheating of the jar. Subsequently, the obtained urea-functionalized P was sieved from the zirconia balls through absolute ethanol. Finally, the resulting dispersion was centrifugated to obtain the sediment, which was then dried overnight in a vacuum oven at room temperature.

### 2.3. Preparation of TiO_2_ Nanoparticles

TiO_2_ nanoparticles were synthesized via the sol–gel method using TTIP precursors. A dilute nitric acid solution was prepared by adding 0.26 mL of nitric acid to 200 mL of distilled water. A total of 2 mL of TTIP was dissolved in 18 mL of absolute ethanol and slowly added dropwise to the dilute nitric acid solution. The mixture solution was stirred at 600 rpm for 12 h at 90 °C. After the reaction, the TiO_2_ powder was collected through vacuum filtration. The resulting powder was finely ground using a mortar and then heat-treated in a furnace at 500 °C to obtain TiO_2_ nanoparticles.

### 2.4. Synthesis of TiO_2_–PPy and P–PPy Nanocomposites

**Figure 1:**
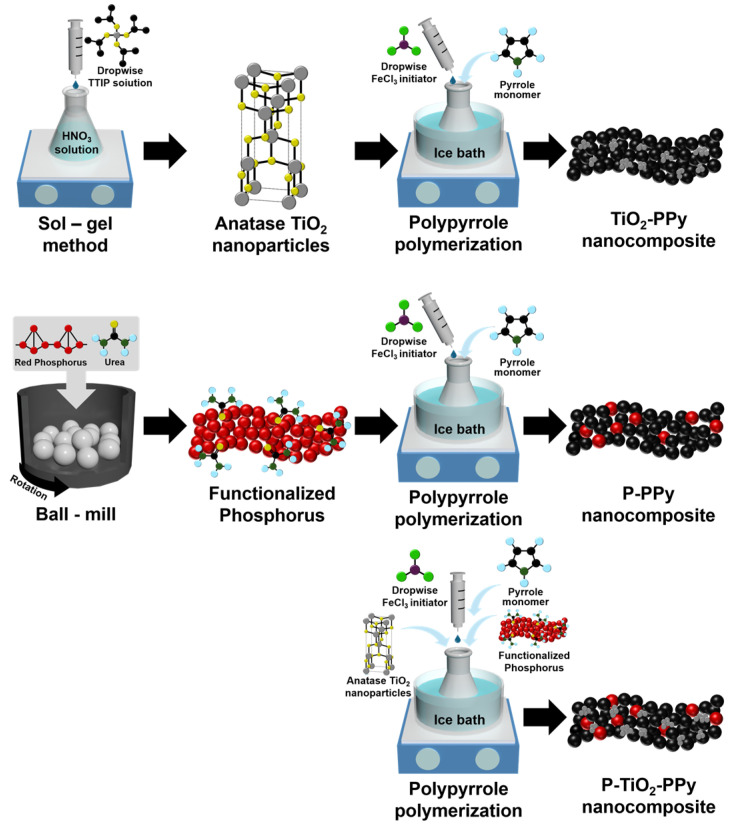
Schematic of the fabrication of TiO_2_–PPy, P–PPy, and P–TiO_2_–PPy nanocomposites for anode nanomaterials via mechanochemical ball milling and sol–gel method, followed by chemical oxidative polymerization.

A total of 1 mL of pyrrole monomer and 0.2 g of TiO_2_ nanoparticles were dispersed in 200 mL of distilled water. An aqueous FeCl_3_ solution was then added dropwise with a monomer-to-oxidant ratio of 1:4, and the mixture was allowed to react for 1 h. To prevent Ostwald ripening of PPy, the solution was stirred for 8 h at a low temperature range of 0–5 °C [19]. Next, the P–PPy nanocomposite was synthesized using the same method as described above for the TiO_2_–PPy nanocomposite, with the only difference being that equivalent amounts of urea-functionalized P replaced the TiO_2_ nanoparticles. After synthesis, the resulting nanocomposites were washed with distilled water to remove any impurities or residual FeCl_3_, and then dried overnight at room temperature in a vacuum oven. The schematic diagram illustrating the experimental procedures for preparing the nanocomposites is shown in Figure 1.

### 2.5. Fabrication of Ternary P–TiO_2_–PPy Nanocomposite

The synthesis of P–TiO_2_–PPy nanocomposites involved dispersing 1 mL of pyrrole monomer, 0.2 g of TiO_2_ nanoparticles, and 0.2 g of urea-functionalized P in 200 mL of distilled water. An aqueous FeCl_3_ solution was then added dropwise and allowed to react for 1 h, with a monomer-to-oxidant ratio of 1:4. The solution was stirred for 8 h at a temperature between 0 and 5 °C to prevent Ostwald ripening of PPy [19]. Following the reaction, P–TiO_2_–PPy nanocomposites were obtained after filtration and washing with distilled water to remove any impurities or residual FeCl_3_. Subsequently, the nanocomposites were dried overnight at room temperature in a vacuum oven.

### 2.6. Characterization

Field emission scanning electron microscopy (FE-SEM; GEMINISEM 300, Carl Zeiss, Oberkochen, Germany) and transmission electron microscopy (TEM; F200s, Talos, Waltham, MA, USA) were utilized to observe the morphological characteristics of the nanocomposites. High-angle annular dark-field scanning transmission electron microscopy (HAADF-STEM; F200s, Talos, Waltham, MA, USA) and energy-dispersive X-ray spectroscopy (EDS; F200s, Talos, Waltham, MA, USA) were employed to confirm element distribution within the nanocomposites. Fourier-transform infrared spectroscopy (FT-IR; VERTEX70, Bruker, Ettlingen, Germany), Raman spectroscopy (Renishaw, Wotton-under-Edge, UK), and X-ray diffraction (XRD; D2 phase, Bruker, Ettlingen, Germany) were utilized to confirm the molecular structure and composition of the synthesized materials. 

### 2.7. Li-Ion Battery Performance

To evaluate the LIB performance of different anode materials, including pure P, pure TiO_2_, P–PPy, TiO_2_–PPy, and P–TiO_2_–PPy, each anode slurry was prepared by mixing the active material (70 wt%), a conductive additive (20 wt%, Super-P), and a binder (10 wt%, poly(vinylidene fluoride) (PVDF)). These component mixtures were thoroughly mixed in a mortar with a pestle for 30 min, followed by the addition of N-methyl-2-pyrrolidone (NMP) solution. The resulting slurry was cast onto a carbon-coated Cu foil current collector (MTI Korea, Seoul, Republic of Korea) using a doctor blade. The slurry cast on the Cu current collector was dried at 80 °C for 12 h. A disk-shaped working electrode was prepared by punching out a hole with a 16 mm diameter. A CR2032 coin cell was assembled in an argon-filled glovebox with humidity and oxygen levels maintained below 0.1 ppm. In the cell, Li metal served as both the counter and reference electrode. A monolayer polypropylene (PP) separator was employed (Celgard 2400, MTI Korea, Seoul, Republic of Korea). The distance between the electrodes in the cell is nearly identical to the 25 µm thickness of the separator. The electrolyte was formulated as 1 M LiPF_6_ in a solvent mixture of ethylene carbonate (EC), ethyl methyl carbonate (EMC), and dimethyl carbonate (DMC) in a volume ratio of 1:1:1 (EC:EMC:DMC) (Soulbrain, Seongnam, Republic of Korea). The LIB performance of the aforementioned anode materials was tested in the coin cell using a multi-channel battery testing system (Neware, Hong Kong, China) at room temperature in galvanostatic mode (constant current density). The cell underwent charge and discharge cycles within a voltage range of 0.01 to 3 V. The C-rate was set at 1C, which corresponds to a full charge or discharge to its theoretical specific capacity within one hour. The theoretical specific capacity is calculated by summing the values obtained from the weight fraction of each component multiplied by its theoretical specific capacity, specifically P (2596 mAh/g) and TiO_2_ (335 mAh/g). Electrochemical impedance spectroscopy (EIS) measurements were conducted on cells after five cycles using an impedance analyzer (ZIVE MP2; Won-A Tech, Seoul, Republic of Korea) at room temperature. For EIS measurement, a sinusoidal AC voltage with a frequency range from 10 mHz to 1 MHz and an amplitude of 5 mV was applied.

## 3. Results and Discussion

### 3.1. Synthesis and Characterization of Anode Materials

#### 3.1.1. Synthesis of Single-Component Anodes

Single-component anode materials, including P, TiO_2_, and PPy, were synthesized through the sol–gel method, mechanochemical ball milling, and chemical oxidative polymerization, respectively, as illustrated in Figure 1. TiO_2_ nanoparticles were synthesized using a sol–gel method, with the dropwise addition of precursor TTIP enabling the achievement of uniform TiO_2_ nanoparticle sizes. The TiO_2_ nanoparticles were sintered to obtain a highly crystalline anatase phase. Pure P anode active materials were synthesized via mechanochemical ball milling of RP with urea additives. Urea was incorporated to introduce functional groups that enhance bonding between P and the polymer chains. The high surface area of RP, along with the urea functional groups grafted onto its surface through the ball mill process, made it possible to form effective P-based nanocomposites [20]. Pure PPy was also synthesized by oxidative polymerization from pyrrole monomer in the presence of FeCl_3_ as an initiator [21]. The morphology and elemental analysis of the pure P, TiO_2_, and PPy were characterized by SEM, TEM, HAADF-STEM, and EDS mappings (Figures S1–S3).

#### 3.1.2. Synthesis and Characterization of PPy-Based Composite Anodes

Inorganic–organic nanocomposites, including TiO_2_–PPy, P–PPy, and P–TiO_2_–PPy nanocomposites, were successfully synthesized by incorporating nanoscale inorganic materials during the polymerization process of PPy. The polymerization from pyrrole monomer was initiated with the dropwise addition of FeCl_3_ solution in the presence of the inorganic materials. One-pot chemical oxidation polymerization offers the advantage of mass-producing ternary nanocomposites (Figure S3).

**Figure 2:**
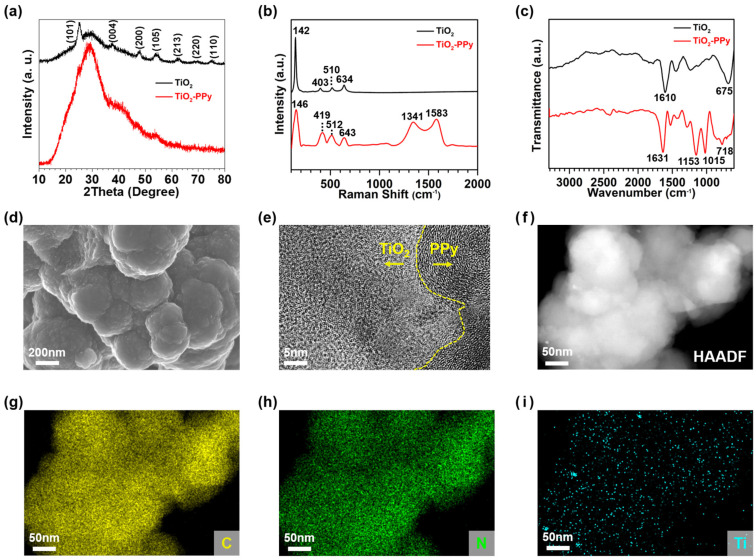
Structural characterization of pure TiO_2_ and TiO_2_–PPy nanocomposites: (**a**–**c**) XRD patterns, Raman spectra, and FT-IR spectra of TiO_2_ and TiO_2_–PPy nanocomposites; (**d**) SEM image, (**e**) TEM image, and (**f**–**i**) HAADF-STEM image and EDS elemental mappings of TiO_2_–PPy nanocomposites.

The TiO_2_ nanoparticles synthesized exhibit the anatase phase, as evidenced by the XRD patterns of pure TiO_2_ (Figure 2a). The crystal structure is confirmed by corresponding peaks of 25°, 38°, 48°, 54°, 63°, 69°, and 75°, which correspond to the (101), (004), (200), (105), (204), (220), and (215) crystal planes of anatase, respectively, according to reference data (JCPDS no. 21-1272) [22]. In contrast, the crystal structure of TiO_2_ in TiO_2_–PPy nanocomposites is challenging to determine from their XRD spectrum due to the absence of distinct peaks (Figure 2a). The negligible XRD peaks may be attributed to the low compositional fraction (~17%) of TiO_2_ in the TiO_2_–PPy anode. The broad XRD peak of the nanocomposite at around 28° could originate from amorphous PPy [23]. To characterize the presence of TiO_2_ in the composite, we analyzed the Raman spectra of the pure TiO_2_ and the TiO_2_–PPy nanocomposites (Figure 2b). Anatase TiO_2_ exhibits characteristic E_g_ peaks at 142 cm^−1^ and 634 cm^−1^, along with B_1g_ and A_1g_ peaks at 403 cm^−1^ and 510 cm^−1^, respectively [24]. These four characteristic peaks are also observed in the Raman spectrum of TiO_2_–PPy at 146 cm^−1^ (E_g_), 419 cm^−1^ (B_1g_), 512 cm^−1^ (A_2g_), and 643 cm^−1^ (E_g_), confirming the presence of anatase TiO_2_ in the organic–inorganic nanocomposites. The Raman spectrum of the TiO_2_–PPy nanocomposites also displays D and G bands at 1341 cm^−1^ and 1583 cm^−1^, respectively. The D band represents the stretching of the aromatic ring in PPy, while the G band represents the π–conjugated C=C bonds of PPy [25]. Furthermore, the FT-IR spectrum (Figure 2c) confirms the presence of TiO_2_, indicated by the two peaks at 675 cm^−1^ and 1610 cm^−1^, corresponding to the bending of Ti–O and the stretching of Ti–OH, respectively [26]. Peaks at 1153 cm^−1^ and 1015 cm^−1^ are characteristic of the bending vibrations of the aromatic ring in PPy [27]. 

Figure 2d displays SEM images of the TiO_2_–PPy nanocomposites, revealing larger particle sizes compared to those of pure TiO_2_ (Figure S1a,b). The surface of the TiO_2_–PPy appears smoother than that of pure TiO_2_, attributed to the thick coating of polymerized PPy on the surface of TiO_2_. To further analyze the morphological features of TiO_2_ and TiO_2_–PPy, we conducted TEM analysis. Pure TiO_2_ nanoparticles show sizes ranging from less than 20 nm (Figure S1c) and an anatase crystalline structure, evident from the lattice pattern in the TEM image (Figure S1d). A TEM image of TiO_2_–PPy nanocomposites reveals crystalline TiO_2_ and amorphous PPy with a layered structure (Figure 2e). The elemental distributions of the nanocomposites are confirmed by EDS element mappings and their corresponding HAADF-STEM image. The elemental mapping clearly exhibits that the nanocomposites are composed of TiO_2_ nanocrystals dispersed in PPy (Figure 2f–i). Therefore, we can confirm that the two-component organic–inorganic TiO_2_–PPy nanocomposite is successfully synthesized by in situ polymerization. 

**Figure 3:**
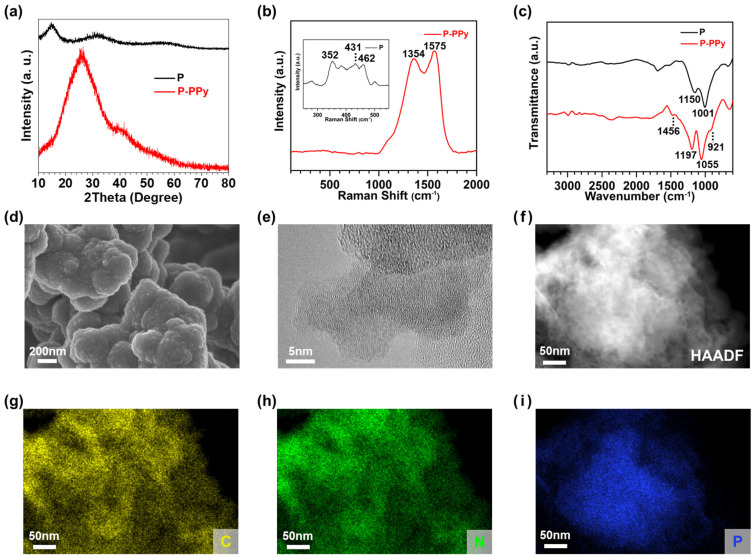
Structural characterization of pure P and P–PPy nanocomposite anode materials: (**a**–**c**) XRD patterns, Raman spectra, and FT-IR spectra of amorphous P and P–PPy nanocomposite; (**d**) SEM image, (**e**) TEM image, and (**f**–**i**) HAADF-STEM image and EDS elemental mappings of P–PPy nanocomposites.

We conducted structure characterization of pure P and P–PPy nanocomposites, as the crystal structures of anodes influence their LIB performance. The XRD spectrum of pure P shows two broad humps at 15 and 32°, indicating that P is in an amorphous state (Figure 3a) [28]. In contrast, the P–PPy composites display a broad characteristic XRD peak at approximately 26°, corresponding to amorphous PPy, without any other P-related peaks [23]. This phenomenon is attributed to the thick coating of PPy on the P in the P–PPy sample. In the Raman spectra (Figure 3b), the characteristic peaks of pure P, specifically the A_1g_, B_2g_, and A_2g_ modes, are observed at 352, 431, and 462 cm^−1^, respectively [29]. In the Raman spectra of P–PPy, the peaks at 1354 cm^−1^ and 1575 cm^−1^ correspond to the D band and G band of PPy, respectively. Because PPy is present in a higher mole fraction than P, the characteristic peaks of PPy are more prominent than those of P [30]. According to the FT-IR spectra (Figure 3c), pure P exhibits peaks at 1001 cm^−1^ and 1150 cm^−1^, which are from the stretching vibration of P–O and P=O, respectively. The dominant characteristic peaks at 1055 cm^−1^ and 1197 cm^−1^ in the IR spectrum of P–PPy correspond to the aromatic-ring bending vibrations of PPy. The peaks observed at 921 cm^−1^ and 1456 cm^−1^ signify the presence of the P–O–C bond and the C=N in-plane vibration of urea in urea-functionalized P, respectively [31]. 

To investigate the morphological features of both pure P and P–PPy nanocomposite anodes, SEM images were analyzed. We observed that the particle size of P–PPy (Figure 3d and Figure S5a) is larger than that of pure P (Figure S2a,b), which is consistent with the findings for pure TiO_2_ and TiO_2_–PPy nanocomposite anodes, as mentioned earlier. Additionally, structural analysis was confirmed through TEM images. Both pure P and P–PPy exhibit amorphous structures, as no distinguishable lattice patterns are observed in the TEM images of pure P (Figure S2c,d) and P–PPy (Figure 3e). From the HAADF-STEM image in Figure 3f, we can interpret that the plane-like morphology of pure P is transformed to a spherical morphology after polymerization of PPy. The successful hybridization of PPy is confirmed by EDS mapping of the P–PPy composites. The elements of C from PPy and P are uniformly distributed across the overall area of P–PPy (Figure 3g–i), indicating that PPy is uniformly incorporated with P.

**Figure 4:**
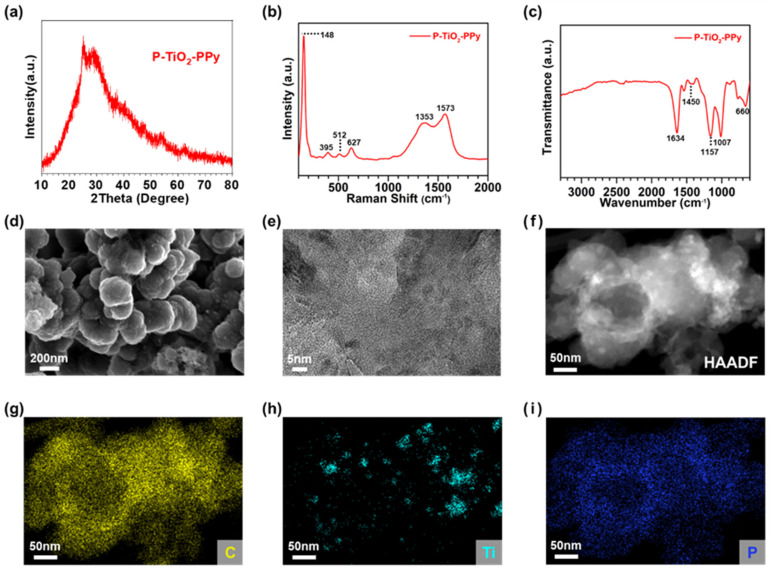
Structural analyses of P–TiO_2_–PPy nanocomposite anode materials: (**a**) XRD pattern, (**b**) Raman spectra, (**c**) FT-IR spectra, (**d**) SEM image, (**e**) TEM image, (**f**) HAADF-STEM image and (**g**–**i**) EDS elemental mappings of P–TiO_2_–PPy nanocomposites.

Noticeably, it can be highlighted that the use of PPy enables fabrication of multi-component nanocomposites by incorporating PPy and two different as-synthesized inorganic materials. To validate this concept, we successfully synthesized P–TiO_2_–PPy nanocomposites via polymerization of pyrrole monomer in the presence of TiO_2_ and P mixture [21,32]. The XRD spectrum of the P–TiO_2_–PPy nanocomposites shows negligible TiO_2_ and P peaks due to the large portion of PPy coating on both TiO_2_ and P (Figure 4a), which differs from the distinguishable peaks of the anatase phase of TiO_2_ (Figure 2a) and broad peaks of amorphous P (Figure 3a). The presence of each component in the ternary composite can be identified by the Raman spectra (Figure 4b). The depicted spectra exhibit distinct peaks corresponding to anatase TiO_2_ at 148, 395, 512, and 627 cm^−1^, representing the E_g_, B_1g_, A_2g_, and E_g_ modes, respectively [24]. Additionally, peaks at 1353 and 1573 cm^−1^ are attributed to the D band and G band of PPy [25]. The prevalence of PPy peaks relative to those of P can be attributed to the higher weight fraction of PPy coating on P compared to pure P. The FT-IR analysis illustrated in Figure 4c reveals the presence of urea-functionalized P in the P–TiO_2_–PPy nanocomposite, as evidenced by the peak observed at 1450 cm^−1^ corresponding to the C=N in-plane vibration from urea that is coated on P [31]. Additionally, two characteristic peaks at 660 cm^−1^ and 1634 cm^−1^ are attributed to the Ti–O bending and Ti–OH stretching in TiO_2_, respectively [26]. Moreover, the two peaks observed at 1007 and 1157 cm^−1^ indicate the aromatic-ring bending vibration of PPy [27]. In Figure 4d, SEM images of P–TiO_2_–PPy illustrate particles varying in sizes from 0.2 to 1 μm. TEM images unveil a more intricate morphology of the three-component nanocomposite of P–TiO_2_–PPy compared to both TiO_2_–PPy and P–PPy (Figure 4e). This intricate morphology can arise from the concurrent presence of small-sized TiO_2_ nanocrystals and plate-like P structures within the PPy network, covering both TiO_2_ and P simultaneously. The HAADF image of the P–TiO_2_–PPy (Figure 4f) displays brighter spots compared to that observed in the P–PPy nanocomposite (Figure 3f). This distinction can be attributed to the presence of TiO_2_ nanoparticles dispersed in the P–TiO_2_–PPy, as verified by the EDS mapping of Ti (Figure 4h). Moreover, C originating from PPy and P are uniformly distributed across the entire area of P–TiO_2_–PPy, indicating the homogeneous coating of PPy onto P (Figure 4g,i).

### 3.2. Li-Ion Battery Performance of PPy-Based Nanocomposite Anodes

#### 3.2.1. Li-Ion Battery Performance of TiO_2_–PPy Anodes

**Figure 5:**
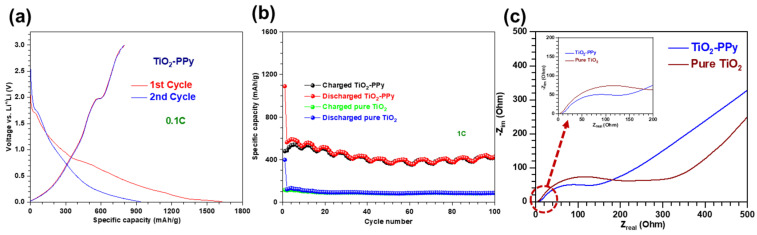
Electrochemical performance of LIBs using both pure TiO_2_ and TiO_2_–PPy anode materials. (**a**) Initial voltage profiles versus specific capacity for TiO_2_–PPy conducted at 0.1C. (**b**) Cycling stability performance of both anode materials. (**c**) EIS measured at the fifth fully charged state.

We investigate the effect of PPy coating on TiO_2_ nanoparticles, a typical conversion-type anode material. Figure 5a illustrates the initial voltage profile versus specific capacity for the TiO_2_–PPy hybrid anode structure. A characteristic Faradic process, by charge transfer at the electrode–electrolyte interface and indicated by a plateau region, is observed at approximately 2 V during the charging process and between 1.5 and 1.7 V during the discharging process. These findings are consistent with those of pure TiO_2_ (Figure S6). The plateau corresponds to the intrinsic capacity values of anatase TiO_2_, originating from lithiation through phase transition from TiO_2_ to Li_0.55_TiO_2_ [33]. Figure 5b demonstrates that the TiO_2_ anode, incorporated with PPy, achieves a specific capacity of 422 mAh/g after 100 cycles, which is nearly 485% higher than that of pure TiO_2_ (87 mAh/g). Additionally, the TiO_2_–PPy anode material demonstrates a stable specific capacity with minimal capacity degradation. The Coulombic efficiency (CE, η) is defined as

$$\eta =\frac{c_{d}}{c_{c}}$$

where *C_d_* is the discharge capacity and *C_c_* is the charge capacity of a half cell in a single cycle [34], which is recorded to be approximately 100%.

The excellent LIB performance of TiO_2_–PPy is strongly related to the role of PPy as an electronic conducting agent [18]. The enhancement of conductivity through PPy incorporation is investigated using electrochemical impedance spectroscopy (EIS) (Figure 5c). The charge transfer resistance between the electrode and electrolyte is measured by the diameter of the semicircle in the high and intermediate frequency range of the EIS spectrum [35,36]. Based on the fitted equivalent circuit shown in the inset of Figure 5c, the charge transfer resistance of TiO_2_–PPy (190 Ω) is lower than that of pure TiO_2_ (219 Ω), indicating faster charge transfer of Li^+^ through the conducting PPy polymer backbones. Solvated Li^+^ ions in the electrolyte are reduced to zero-valent Li by electron transfer from the electrode. The resistance resulting from the charge transfer process is denoted as R_ct_, which results from contributions from both the desolvation step and the diffusion in the SEI step. The R_ct_ follows a relationship based on a thermally activated process as follows:

$$\frac{1}{R_{ct}}=A_{0}\exp(-\frac{E_{a}}{RT})$$

where *A*_0_, *E_a_*, *R*, and *T* are a frequency factor, the activation energy, the gas constant, and the temperature in Kelvin, respectively. The *E_a_* of the Li^+^ charge transfer process represents the barrier that the Li^+^ needs to overcome to cross the interface between the electrolyte and the electrode. The value of E_a_ can be obtained from the slope of a log(1/*R_ct_*) versus the inverse of the temperature (1/*T*) plot [37]. Therefore, PPy can contribute to the enhanced charge transfer because of the lower activation energy, consequently facilitating Li^+^ uptake within a fixed time to yield higher specific capacity.

#### 3.2.2. Li-Ion Battery Performance of P–PPy

**Figure 6:**
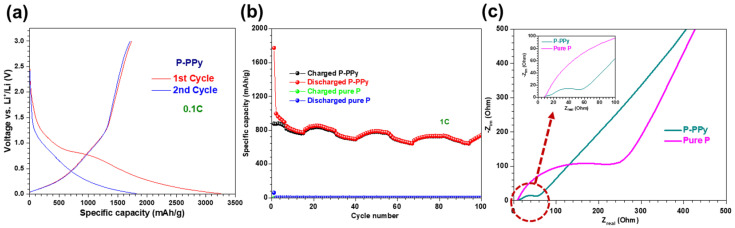
Electrochemical performance of a LIB using pure P and P–PPy anode materials. (**a**) Initial voltage profiles versus specific capacity for P–PPy conducted at 0.1C. (**b**) Cycling stability of both anode materials. (**c**) EIS results measured at the 100th fully charged state.

We also investigate the effect of PPy coating on P, which serves as a typical intercalation-type anode. The initial two voltage profiles, which vary with specific capacity, show a gradual increase in voltage from 1.0 V to 1.2 V during the charging process (Figure 6a). The continuous capacity increase without a plateau region can be attributed to the absence of a phase transition at a specific voltage as the lithiation of amorphous P occurs via the intercalation mechanism [2]. In the voltage profile of the first cycle, the plateau region at approximately 0.8 V indicates SEI formation due to electrolyte decomposition, causing a low CE of ~54%. In contrast, the CE in the second cycle is nearly 100%. Figure 6b shows that the P–PPy anode achieves a specific capacity of 741 mAh/g after 100 cycles, accompanied by ~100% of CE. This specific capacity is four orders of magnitude higher than that of pure P, which is only 0.07 mAh/g (refer to green and blue spheres in Figure 6b) due to its low conductivity [2]. The origin of the exceptional LIB performance is similar to that of the LIB performance of TiO_2_–PPy, attributed to the electronic conducting effect [18]. EIS characterization indicates that the charge transfer resistance of PPy-incorporated P (62.6 Ω) is lower than that of pure P (325 Ω) (see Figure 6c). As discussed earlier, the relationship between charge transfer resistance and activation energy suggests that PPy enhances the specific charge capacity of P [18]. Compared to pure P, the P–PPy sample exhibits a slope closer to a 45° angle, indicating a relationship with Warburg impedance [38]. These results confirm that P–PPy facilitates more efficient mass transfer of Li^+^ ions in its anode structure than amorphous pure P, which has lower electrical conductivity. This notable improvement is mainly due to the influence of PPy on enhancing the electrical conductivity of amorphous pure P [18].

#### 3.2.3. Li-Ion Battery Performance of P–TiO_2_–PPy

**Figure 7:**
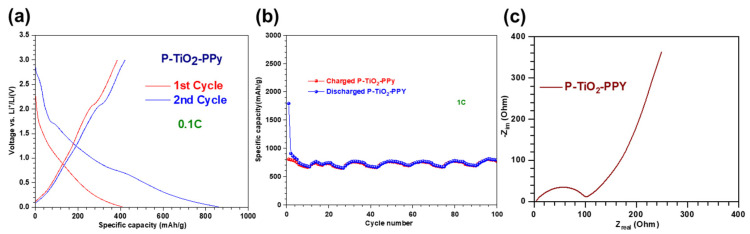
Electrochemical performance of a LIB using P–TiO_2_–PPy anode material. (**a**) Initial voltage profiles versus specific capacity for P–TiO_2_–PPY conducted at 0.1C. (**b**) Cycling stability performance of the P–TiO_2_–PPy anode sample. (**c**) EIS results measured at the 100th fully charged state.

Integrating PPy into both intercalation- and conversion-type anode materials provides a proof-of-concept for synthesizing three-component nanocomposite anodes, including P–TiO_2_–PPy. We examine the electrochemical properties of P–TiO_2_–PPy to evaluate their potential for use in LIB anodes. Initial voltage profiles corresponding to the variation in specific capacity for the three-component P–TiO_2_–PPy anode materials are illustrated in Figure 7a. During the first cycle, plateau regions are displayed at ~2 V for the charging process and ~1.7 V for the discharging process. These plateau regions can correspond to the intrinsic capacity values of anatase TiO_2_, resulting from the lithiation-induced phase transition from TiO_2_ to Li_0.55_TiO_2_ [33]. Conversely, the gradual increase in capacity below 1.7 V, without a plateau, can be attributed to the lack of a phase transition at a specific voltage, as lithiation of amorphous P occurs via an intercalation mechanism [2]. These findings are consistent with our data shown in Figure 5a and Figure 6a, along with previous reports [30,33]. In the voltage profile of the first cycle, the plateau region at approximately 0.8 V indicates SEI formation due to electrolyte decomposition, resulting in a low CE of approximately 49%. In contrast, the CE in the second cycle is nearly 94%. Figure 7b shows that the P–TiO_2_–PPy anode reaches up to a specific capacity of 790 mAh/g after 100 cycles. As can be seen in Figure 7c, EIS data indicate that the charge transfer resistance (R_ct_) of P–TiO_2_–PPy (106.4 Ω) is between the results of PPy-based hybrid anodes (TiO_2_–PPy (40.6 Ω) and P–PPy (62.6 Ω)) and those of pure anodes (pure TiO_2_ (219 Ω) and pure P (325 Ω)), as mentioned earlier. Based on these data, we may believe that P–TiO_2_–PPy possesses a higher activation energy, proportional to the value of R_ct_, compared to those of P–PPy and TiO_2_–PPy [37].

#### 3.2.4. Long-Term Stability of PPy-Based Composite Anodes

**Figure 8:**
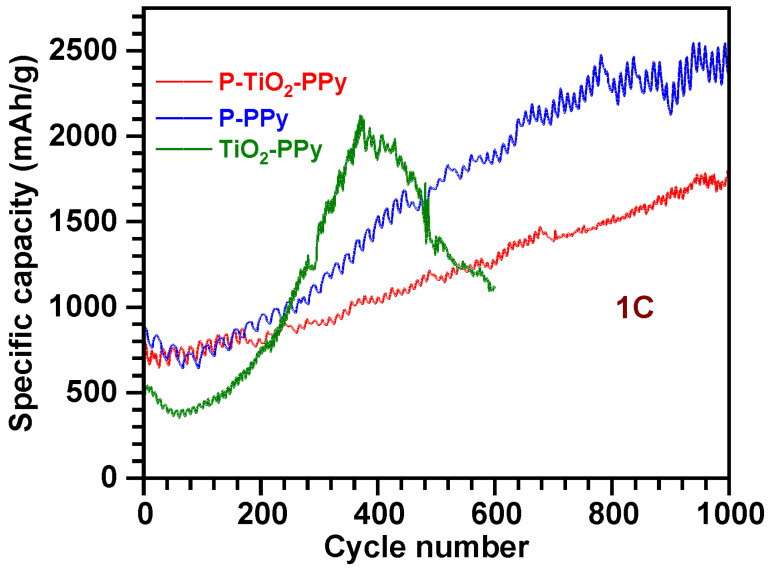
Comparison of cycling stability of the three different samples of LIBs using P–TiO_2_–PPy, P–PPy, and TiO_2_–PPy anode materials under the condition of 1C.

For the industrial implementation of LIBs, it is essential to analyze the long-term cycling stability performance. To this end, we conducted charge and discharge cycling tests using three different anode materials for 500 cycles. Long-term cycling stability data reveal that the specific capacity tends to increase with the cycle number for the three types of PPy-based composite anodes: TiO_2_–PPy, P–PPy, and P–TiO_2_–PPy (Figure 8). The increasing trend may be attributed to the reactivated three-dimensional network structures of PPy for more Li^+^ ion uptake for TiO_2_ nanoparticles and P embedded with PPy. The specific capacities increase to 2123 mAh/g, 1700 mAh/g, and 1168 mAh/g, for TiO_2_–PPy, P–PPy, and P–TiO_2_–PPy, respectively. For the TiO_2_–PPy anode, the long-term cycling stability deteriorates after 371 cycles. This degradation can be attributed to the disintegration of TiO_2_ nanoparticles from the PPy networks and structural pulverization caused by the significant volume change in the TiO_2_ nanoparticles during the Li^+^ ion conversion–deconversion process [39]. The P–PPy anode also demonstrates stable specific capacity with minimal decay. This stability is primarily due to the structural buffering effect of PPy on the P. The mechanically flexible PPy can accommodate a ~490% volume change in the P structure during repeated intercalation–deintercalation reactions with Li^+^ ions. This suggests that PPy can prevent structural pulverization in the P–PPy hybrid anode and maintain its structural integrity throughout prolonged cycling [40]. Additionally, PPy can form strong chemical bonds with P, avoiding direct contact between P and the electrolyte and thereby mitigating the shuttle effect of Li_x_P [3]. Minimizing the formation of Li_x_P also leads to stabilizing the specific capacity with large cycle numbers. 

The P–TiO_2_–PPy nanocomposite anode exhibits remarkable capacity stability, with a steadily increasing specific capacity reaching up to 1763 mAh/g over 1000 cycles, and maintaining a CE of nearly 100% throughout this period (refer to Figure S7). Similar to the previously discussed TiO_2_–PPy and P–PPy nanocomposite anode materials, the electrically conductive and structurally flexible PPy can enhance the electrical conductivity of both P and TiO_2_ active materials and avoid structural degradation caused by volume changes during extended charge and discharge cycles. Additionally, PPy prevents direct contact between the active materials and the electrolyte, thereby circumventing side reactions at the solid electrolyte interface (SEI) [41]. PPy can also form strong bonds with P, mitigating the shuttle effect of Li_x_P, which contributes to the excellent long-term cycling performance [3]. Therefore, we propose that the PPy-based multi-component anode system holds significant promise for advanced Li-ion batteries requiring superior cycling performance.

## 4. Conclusions

P and TiO_2_, known for their high specific capacities, have been extensively investigated as anode materials for LIBs. Nevertheless, P has inherent weaknesses, including poor electrical conductivity (~10^−10^ S/cm) and significant volume variation (~490%) during charge and discharge cycles, leading to structural degradation and reduced capacity during long-term cycling. Similarly, TiO_2_ has limitations of low electrical conductivity (~10^−12^ S/cm) and slow Li^+^ ion diffusion. To address these issues, we synthesized hybrid anode materials by incorporating P with PPy and TiO_2_ with PPy through pyrrole monomer polymerization. Compared to pure P and TiO_2_, these hybrid materials exhibit enhanced cycling stability and specific capacity, primarily due to the conductive and flexible nature of PPy, which improves the electrical conductivity of the anode material and mitigates volume changes during charging and discharging cycles. Notably, we demonstrated that PPy can also form a multi-component nanocomposite anode of P–TiO_2_–PPy, showing a capacity increase up to 1763 mAh/g over 1000 cycles.

## Data Availability

Data are contained within the article and Supplementary Materials.

## Supplementary Materials

The following supporting information can be downloaded at: https://www.mdpi.com/article/10.3390/nano14131138/s1, Figure S1. (a) Low-magnification and (b) high-magnification SEM images of pure TiO_2_ nanoparticles. (c) Low-magnification and (d) high-magnification TEM images of pure TiO_2_ nanoparticles. Figure S2. Morphological characteristics of pure P: (a) The low-magnification SEM image and (b) the high-magnification SEM image of pure P. (c) The low-magnification TEM image and (d) the high-magnification TEM image of pure P. Figure S3. (a) Low-magnification TEM image and (b) high resolution TEM (HRTEM) image of assynthesized PPy. (c) High angle annular dark field-scanning transmission electron microscopy (HAADF-STEM) image and (d,e) EDS images of as-synthesized PPy. Figure S4. (a) Low-magnification SEM image and (b) Low-magnification TEM images of TiO_2_-PPy nanocomposite. (c) HAADF-STEM image and energy dispersive X-ray spectroscopy (EDS) element mapping image of TiO_2_. Figure S5. (a) SEM image and (b) TEM image of P-PPy nanocomposite. Figure S6. Initial voltage profiles versus specific capacity for TiO_2_-PPy conducted at 0.1C. Figure S7. Long-term cycling performance of LIB using P-TiO_2_-PPy nanocomposite anode materials measured at 1C for up to 1000 cycles.
