# Supplementary material for: Influence of Polypyrrole on Phosphorus- and TiO2-Based Anode Nanomaterials for Li-Ion Batteries
Source: Nanomaterials (Basel). 2024 Jul 2;14(13):1138. doi: 10.3390/nano14131138 (PMC11243682; doi:10.3390/nano14131138)
Supplement: Supplementary file 1 [file nanomaterials-14-01138-s001.zip › nanomaterials-3070094-supplementary.pdf]

Supplementary Data

Influence of Polypyrrole on Phosphorus- and TiO_2-Based Anode Nanomaterials for Li-Ion Batteries

Chiwon Kang [1,†], Kibum Song [1,†] , Seungho Ha [1,†] , Yujin Sung [1], Yejin Kim [1] and Keun-Young Shin [1,2,*] and Byung Hyo Kim [1,2,*]

[1] Department of Materials Science and Engineering, Soongsil University, Seoul 06978, Republic of Korea; cwkang@ssu.ac.kr (C.K.)

[2] Department of Green Chemistry and Materials Engineering, Soongsil University, Seoul 06978, Republic of Korea

* Correspondence: skykek@ssu.ac.kr (K.-Y.S.); byunghyokim@ssu.ac.kr (B.H.K.); Tel.: +82-2-829-8217 (K.-Y.S.); +82-2-829-8218 (B.H.K.)

† These authors contributed equally to this work.

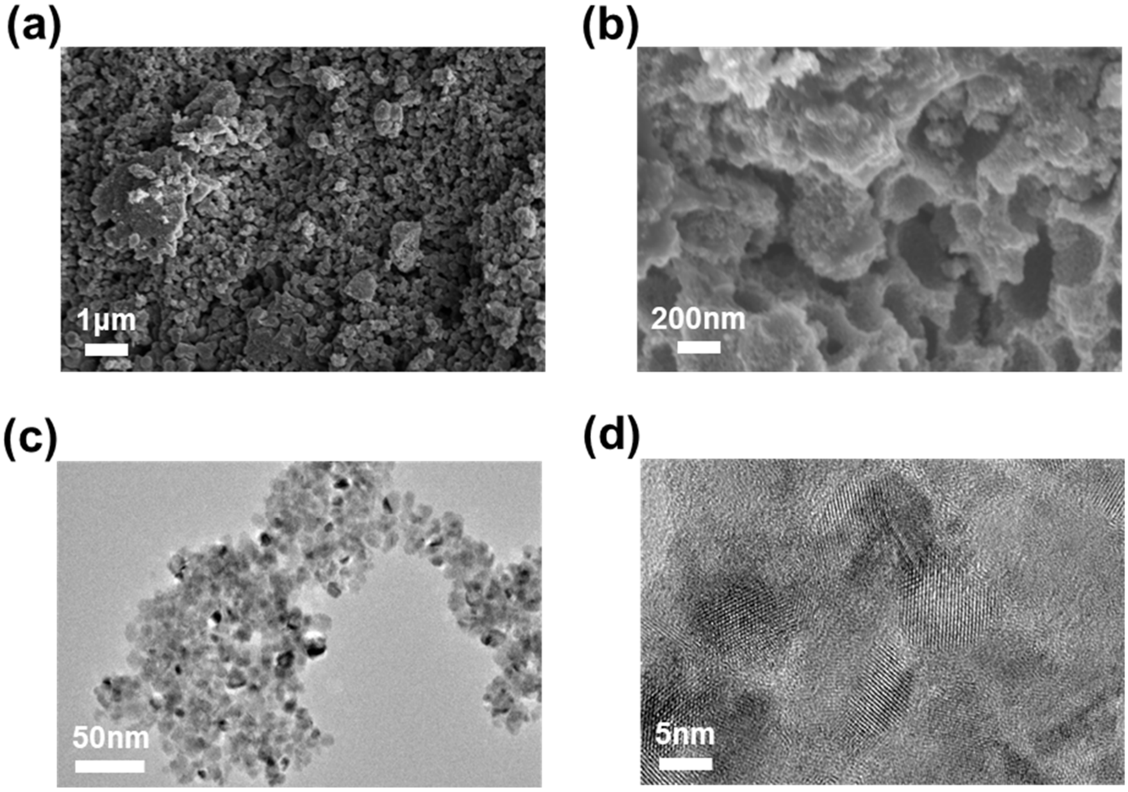

Figure S1. (a) Low-magnification and (b) high-magnification SEM images of pure TiO_2 nanoparticles. (c) Low-magnification and (d) high-magnification TEM images of pure TiO_2 nanoparticles.

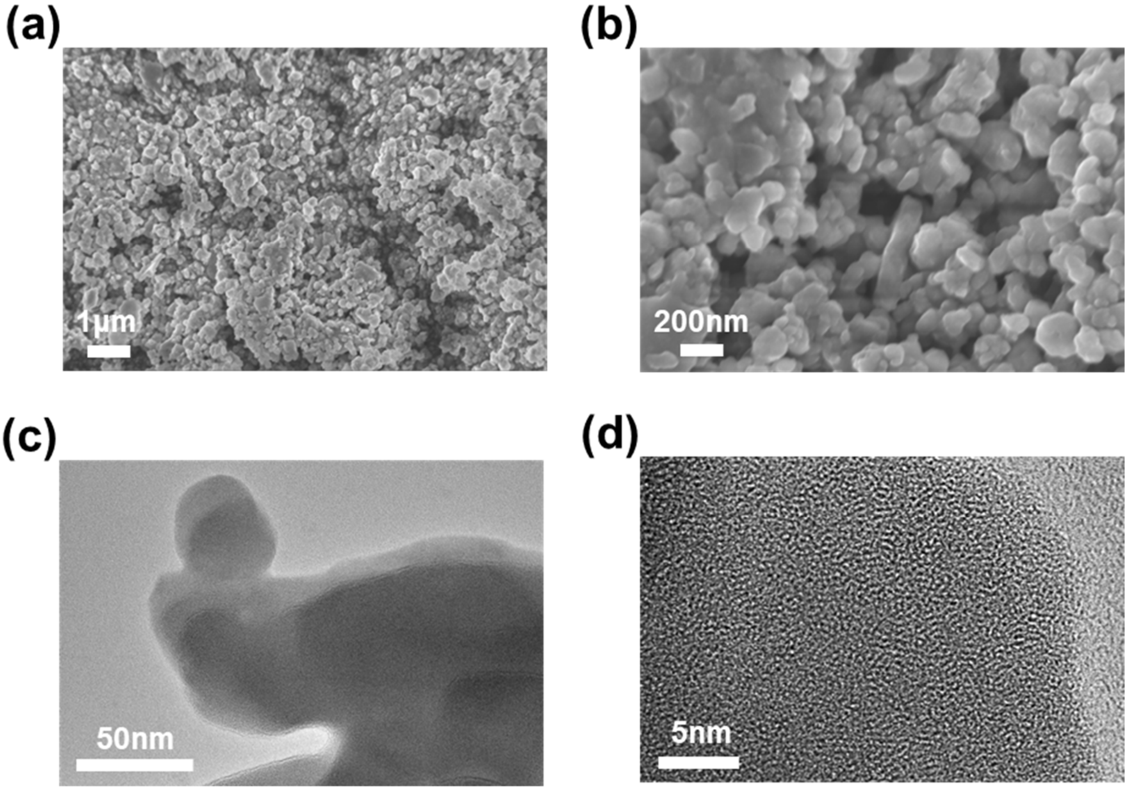

Figure S2. Morphological characteristics of pure P: (a) The low-magnification SEM image and (b) the high-magnification SEM image of pure P. (c) The low-magnification TEM image and (d) the high-magnification TEM image of pure P.

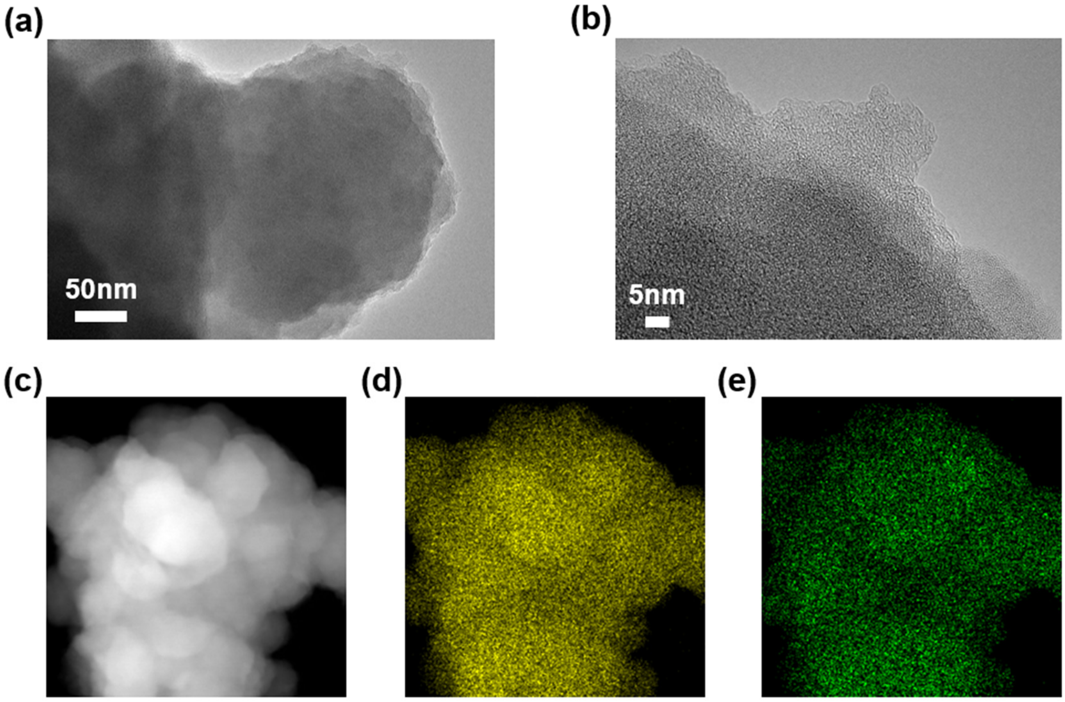

Figure S3. (a) Low-magnification TEM image and (b) high resolution TEM (HRTEM) image of as- synthesized PPy. (c) High angle annular dark field-scanning transmission electron microscopy (HAADF-STEM) image and (d-e) EDS images of as-synthesized PPy.

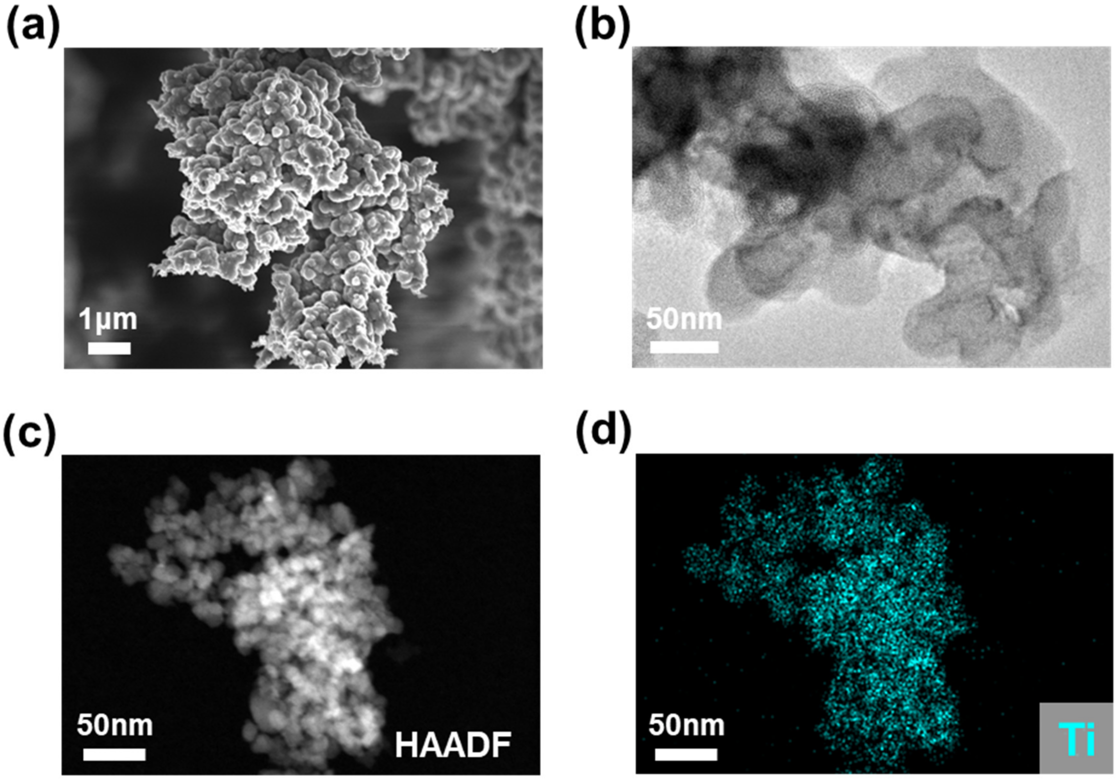

Figure S4. (a) Low-magnification SEM image and (b) Low-magnification TEM images of TiO_2-PPy nanocomposite. (c) HAADF-STEM image and (d) energy dispersive X-ray spectroscopy (EDS) element mapping image of TiO_2.

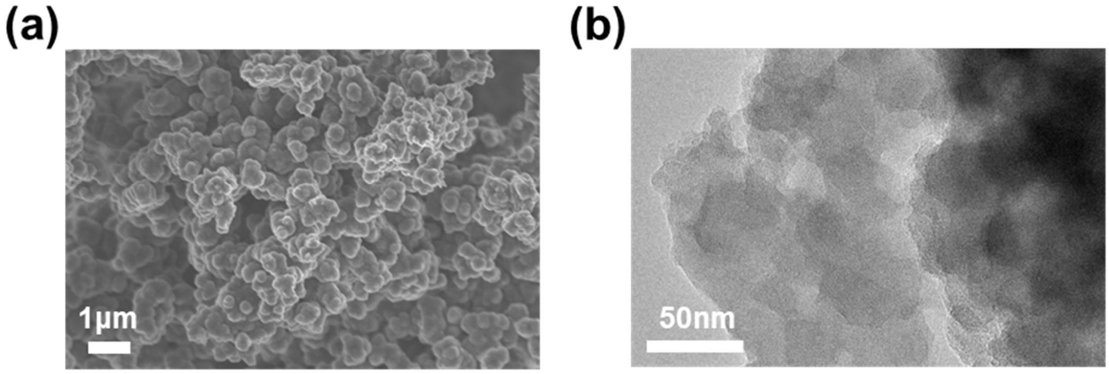

Figure S5. (a) SEM image and (b) TEM image of P-PPy nanocomposite.

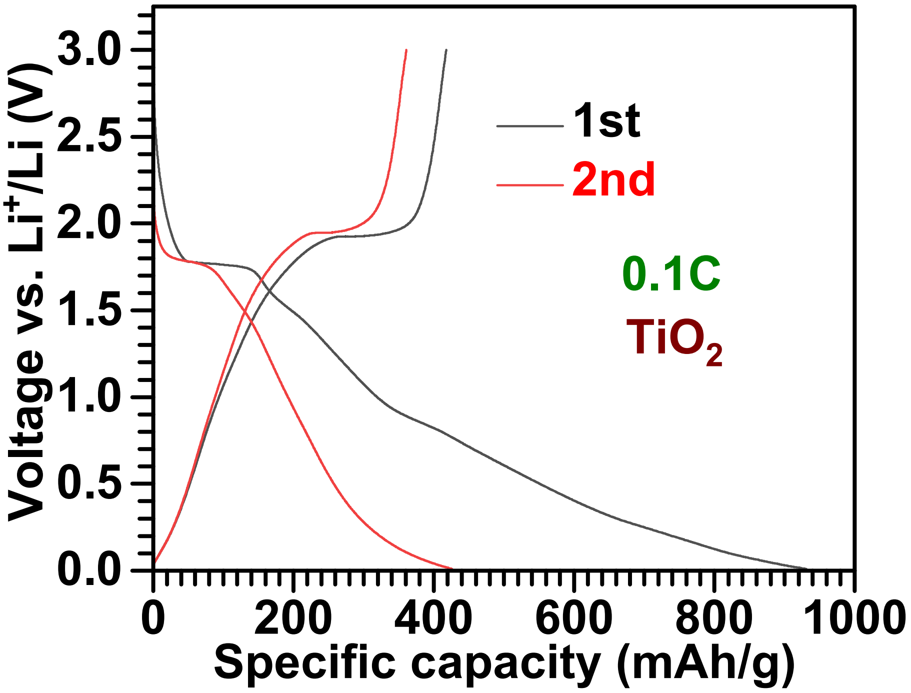

Figure S6. Initial voltage profiles versus specific capacity for TiO_2-PPy conducted at 0.1C.

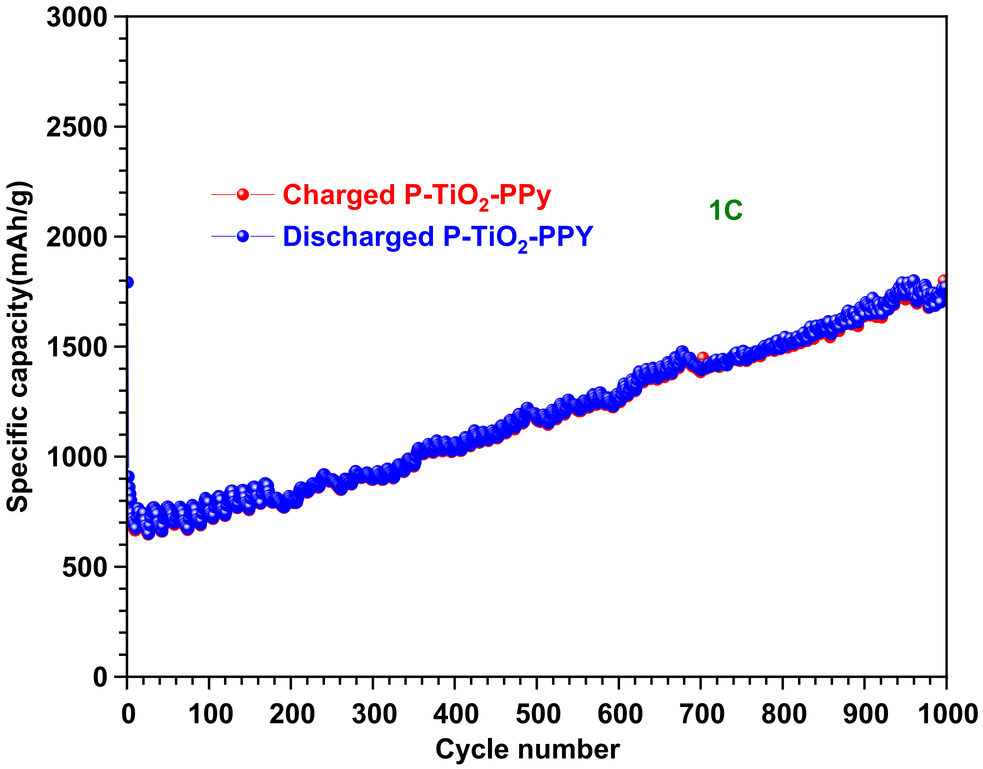

Figure S7. Long-term cycling performance of LIB using P-TiO_2-PPy nanocomposite anode materials measured at 1C for up to 1,000 cycles.
